# *Biochemia Medica* appoints new Editor-in-Chief and new Senior Editor

**DOI:** 10.11613/BM.2018.010301

**Published:** 2018-02-15

**Authors:** Daria Pašalić

**Affiliations:** 1 Department of Medical Chemistry, Biochemistry and Clinical Chemistry, University of Zagreb, School of Medicine, Zagreb, Croatia; 2 Editor-in-Chief, *Biochemia Medica*, Zagreb, Croatia

**Keywords:** editor, journal, scientific writing, publication

Professor Ana-Maria Simundic was the Editor-in-Chief of the *Biochemia Medica* journal from 2011 to 2017, in its „golden era” and expansion of its quality. It was a great experience and pleasure to have learned from her and to have been a part of such a successful development of our Journal. I would like to thank her for giving me the opportunity to continue as the new appointed Editor-in-Chief. I do also thank the Croatian Society for Medical Biochemistry and Laboratory Medicine’s (CSMBLM) Executive Board and the Editorial board of the *Biochemia Medica* for their support.

Each year of Prof. Simundic’s mandate was marked by significant efforts to improve the Journal quality and make it internationally recognizable. The unavoidable result of such a dedicated commitment is the placement of the *Biochemia Medica* at the top position among some of the most esteemed Croatian scientific journals and in the first quartile within the Medical laboratory technology category of the Journal Citation Report (Clarivate Analytics) (*1*, *2*).

Her aim to promote the journal was fully successful. In the first year of her mandate (2011) the Journal already met the inclusion criteria and has been accepted for indexing in the National Library of Medicine/Medline bibliographic database since the beginning of 2012 (*3*, *4*). The next step was to implement responsible research integrity policies and procedures, which in 2012 resulted with the appointment of a Research Integrity Editor and the introduction of a new Journal section called Research integrity corner from the beginning of 2013 (*5*, *6*). *Biochemia Medica* has thus become a journal with high ethical standards. Parallel to this, Prof. Simundic has also initiated the implementation of a regular Cross-Check plagiarism detection and analysis of all submitted manuscripts as a routine procedure within the editorial manuscript processing. Since then *Biochemia Medica* has been accepted for indexing in PubMed Central database in 2014, and in Current Contents database in 2015, thus recognizing our Journal as fulfilling all necessary criteria to be cited in these most prestigious scientific databases in biomedicine (*7*).

I would like also to emphasize the irreplaceable role of former and current Journal editors, as well reviewers and authors and their merits in the successful expansion of the *Biochemia Medica* journal.

Therefore I would like to thank Prof Simundic for everything she has done. In the years of her mandate she was a driving force continuously dedicated to the Journal growth. To honour her merits, I have appointed her as the Journal Senior Editor. I’m convinced she still has a lot of energy, enthusiasm and fresh ideas to support our Journal to further flourish and grow in relevance, impact and recognition.

I’ll do my best to apply all that I have learned from her and I will strive to continue along the same path. I’m sure that this will be possible because of successful and confident team that I have in the Journal Editorial Board.
